# RESOLFT Nanoscopy of Fixed Cells Using a Z-Domain Based Fusion Protein for Labelling

**DOI:** 10.1371/journal.pone.0136233

**Published:** 2015-09-16

**Authors:** Peter Ilgen, Tim Grotjohann, Daniel C. Jans, Markus Kilisch, Stefan W. Hell, Stefan Jakobs

**Affiliations:** 1 Department of NanoBiophotonics, Max Planck Institute for Biophysical Chemistry, Göttingen, Germany; 2 Department of Neurology, University Medical Center of Göttingen, Göttingen, Germany; 3 Department of Molecular Biology, University Medical Center of Göttingen, Göttingen, Germany; University of Manchester, UNITED KINGDOM

## Abstract

RESOLFT super-resolution microscopy allows subdiffraction resolution imaging of living cells using low intensities of light. It relies on the light-driven switching of reversible switchable fluorescent proteins (RSFPs). So far, RESOLFT imaging was restricted to living cells, because chemical fixation typically affects the switching characteristics of RSFPs. In this study we created a fusion construct (FLASR) consisting of the RSFP rsEGFP2 and the divalent form of the antibody binding Z domain from protein A. FLASR can be used analogous to secondary antibodies in conventional immunochemistry, facilitating simple and robust sample preparation. We demonstrate RESOLFT super-resolution microscopy on chemically fixed mammalian cells. The approach may be extended to other super-resolution approaches requiring fluorescent proteins in an aqueous environment.

## Introduction

As described by Ernst Abbe in 1873, diffraction limits the resolving power of conventional far-field optical microscopy to about 200 nm in the optical plane [1,2]. Over the last decade, several optical microscopy techniques (super-resolution microscopy or nanoscopy) have overcome the diffraction barrier, improving the attainable spatial resolution substantially [3,4]. In these approaches, the diffraction limit is overcome by forcing nearby features to fluoresce sequentially [3]. In RESOLFT (***re***versible ***s***aturable ***o***ptica***l f***luorescence ***t***ransition) and the related STED (***st***imulated ***e***mission ***d***epletion) super-resolution microscopy, typically a doughnut or a line pattern is scanned across the sample, determining the nanosized coordinate range where the fluorophores are in their fluorescent On-states [5]. These concepts require a mechanism for reversibly silencing some of the fluorophores that are exposed to the excitation light.

In RESOLFT nanoscopy this is achieved by relying on the light driven switching of reversible switchable fluorescent proteins (RSFPs). RSFPs are structurally similar to the green fluorescent protein (GFP), but may be reversibly photoswitched between metastable fluorescent On- and non-fluorescent Off-states by irradiation with two different wavelengths [6]. Several RSFPs have been successfully used for RESOLFT super-resolution microscopy [7–12].

In RSFP-based RESOLFT nanoscopic imaging, the employed light intensities (typically 1–80 kW/cm^2^) are up to a million times lower than in STED nanoscopy and the total light dose impinging on the cell is typically lower by 3–4 orders of magnitude compared to the stochastic single-molecule based approaches [10]. The intensities used are comparable to those applied in live-cell confocal fluorescence microscopy. Thus RESOLFT microscopy is the super-resolution concept that uses the lowest overall light dose to overcome the diffraction barrier fundamentally. However, photoswitching of RSFPs requires an aqueous environment and chemical fixation of the RSFPs generally deteriorates the light driven switching [13,14]. Presumably for this reason, all biological RESOLFT studies published so far relied on living cells.

In this study, we generated a tool that facilitates RESOLFT imaging on chemically fixed mammalian cells. To this end, we made use of the Z-domain, a 58 amino acid long synthetic protein domain, derived from the *Staphylococcus aureus* cell wall protein A [15]. The Z-domain binds to IgGs of various origin with high affinities [15–17] and has been used for numerous applications including affinity chromatography, immunoprecipation, and others [17,18]. Protein engineering of the Z-domain resulted in the development of small binders (so-called affibodies) with affinities for various targets [19]. Here, we generated fusions of the RSFP rsEGFP2 and the divalent form of the Z domain. The recombinant fusion proteins were purified and used for immunolabelling, allowing RESOLFT super-resolution microscopy on fixed cell samples.

## Results

### Generation of the ZZ-domain-rsEGFP2 fusion protein FLASR

**Fig 1:**
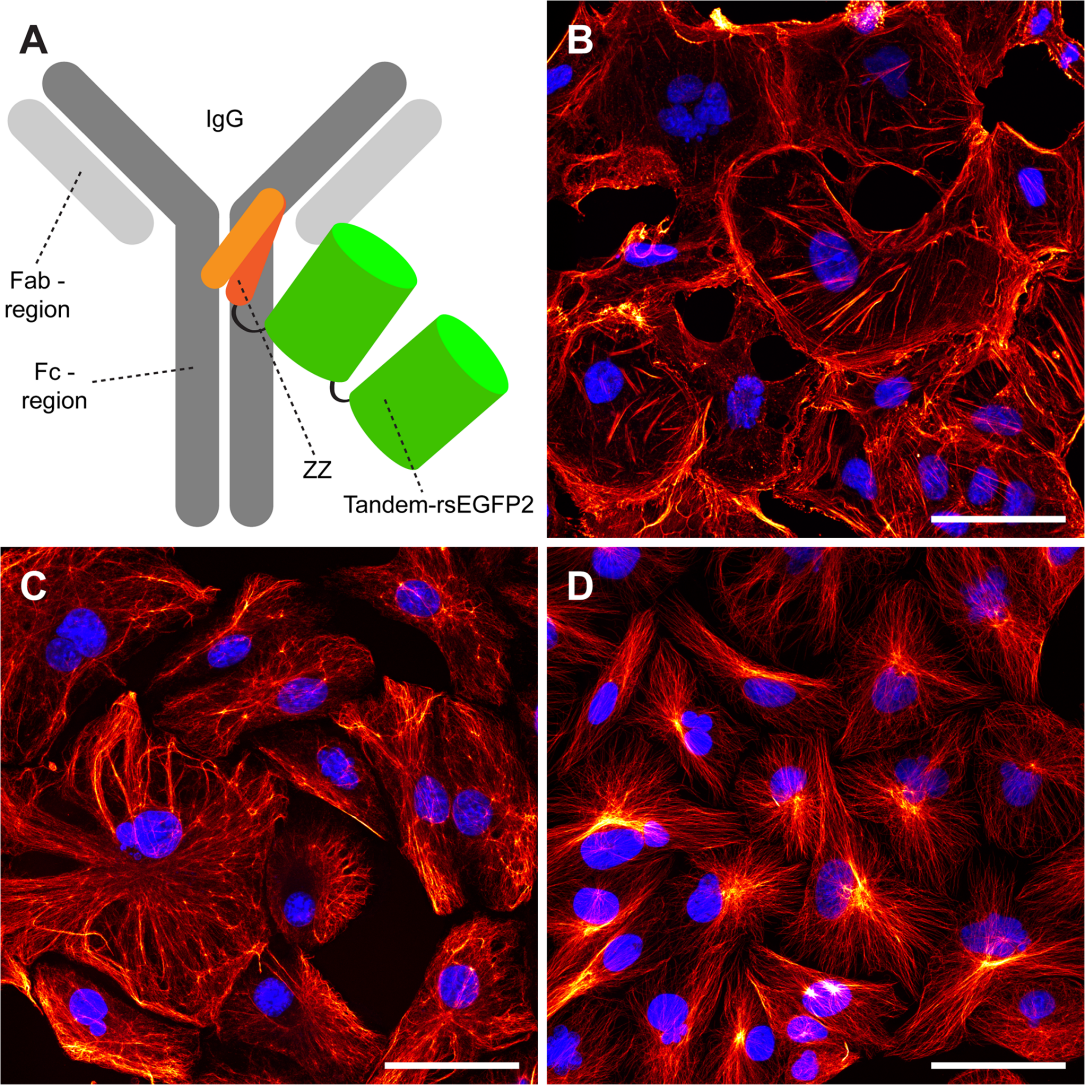
Immunolabelling with FLASR. (A) Schematic of FLASR (ZZ-rsEGFP2_tandem_) bound to an immunoglobulin protein. (B-D) Maximum intensity projections of confocal z-stacks of methanol fixed mammalian CV-1 cells immunolabelled with antibodies against β-actin (B), vimentin (C) and α-tubulin (D). Subsequently, purified FLASR (red) was used to decorate the primary antibodies. Nuclei were stained with DAPI (blue). Scale bars: 50 μm.

The RSFP rsEGFP2 has been demonstrated to be a powerful probe for RESOLFT nanoscopy [10]. As in all RSFPs, chemical fixation affects the switching characteristics of rsEGFP2 and the best light-driven switching performance requires an aqueous environment. In order to generate an rsEGFP2-based probe that facilitates RESOLFT imaging of chemically fixed cells analogous to classical immunofluorescence labelling, we generated a fusion construct consisting of rsEGFP2 and the divalent form of the Z domain (ZZ) that binds the Fc region of IgGs with high affinity [16]. To ensure a strong fluorescence signal, we fused two rsEGFP2 proteins C-terminally to a ZZ domain (Fig 1A). The final construct was named FLASR for ***f***luorescent protein ***l***abelled ***a***ntibody-binder for ***s***uper-***r***esolution microscopy.

The His_6_-tagged FLASR was expressed in *E*. *coli* cells and purified to homogeneity by Ni-NTA affinity chromatography. To test if FLASR maintains the binding specificity of the ZZ-domain to IgGs, we performed Surface Plasmon Resonance spectroscopy to determine the affinity of FLASR to several IgG subspecies that are often used for immunofluorescence labelling. To this end, FLASR was immobilized on a Ni-NTA chip and polyclonal antibody-solutions derived from mouse, rabbit, sheep and goat were injected over the chip surface and association and dissociation of each antibody were recorded in real-time. All tested antibodies showed specific interactions with the immobilized FLASR on the chip surface, with binding affinities between 0.35 ± 0.05 nM and 159 ± 33 nM (Table 1, S1 Fig). The dissociation for all antibodies tested was strongly delayed, which is in agreement with previously reported binding behaviour of the ZZ-domain to human IgGs [16]. We conclude that FLASR binds a range of IgGs with high affinity and specificity.

**Table 1:** Binding parameters of the interaction between FLASR and polyclonal secondary antibody solutions of different mammalian species. FLASR was immobilized on a Ni^2+^-chelator sensor chip. Concentrations of 7.8 nM to 4.0 μM of the indicated IgG were used to monitor the association and dissociation. Data were analyzed with a simple 1:1 Langmuir interaction model to determine rate constants for the association and dissociation, which were then used to calculate the indicated dissociation constants.

	Kinetic parameters		
	k_a_ [M^-1^s^-1^]	k_d_ [s^-1^]	K_D_ [nM]
mouse IgG	1.50 x 10^4^ ± 4.53 x 10^2^	7.70 x 10^−4^ ± 2.73 x 10^−6^	51.36 ± 1.69
rabbit IgG	2.22 x 10^5^ ± 1.32 x 10^4^	7.61 x 10^−5^ ± 6.96 x 10^−6^	0.35 ± 0.05
sheep IgG	5.41 x 10^3^ ± 6.81 x 10^2^	7.96 x 10^−4^ ± 6.84 x 10^−5^	159.40 ± 32.72
goat IgG	1.31 x 10^4^ ± 5.35 x 10^3^	2.62 x 10^−4^ ± 1.63 x 10^−5^	43.18 ± 18.89

### Fluorescence microscopy with FLASR

To investigate the potential of FLASR for immunofluorescence labelling, we decorated methanol fixed mammalian CV-1 cells with primary monoclonal mouse antibodies against β-actin, vimentin, or α-tubulin. Methanol fixation was chosen, because the antibodies used decorate the cytoskeletal elements in methanol fixed cells more reliably than in formaldehyde fixed cells. After a washing step, the cells were decorated with FLASR (0.1 mg/ml) to label the primary antibodies. Subsequently, the cells were mounted in a DAPI containing medium to highlight the nuclei. For confocal imaging, the cells were first imaged with light of 405 nm to visualize the nuclei and to simultaneously switch the rsEGFP2 molecules into their fluorescent On-states. Then, the rsEGFP2 fluorescence was imaged by using excitation light of 488 nm. We found that all three cytoskeletal structures were labelled with high specificity, resulting in images with a very good signal-to-noise ratio comparable to conventional immunofluorescence imaging (Fig 1B–1D).

### RESOLFT nanoscopy of fixed cells labelled with FLASR

**Fig 2:**
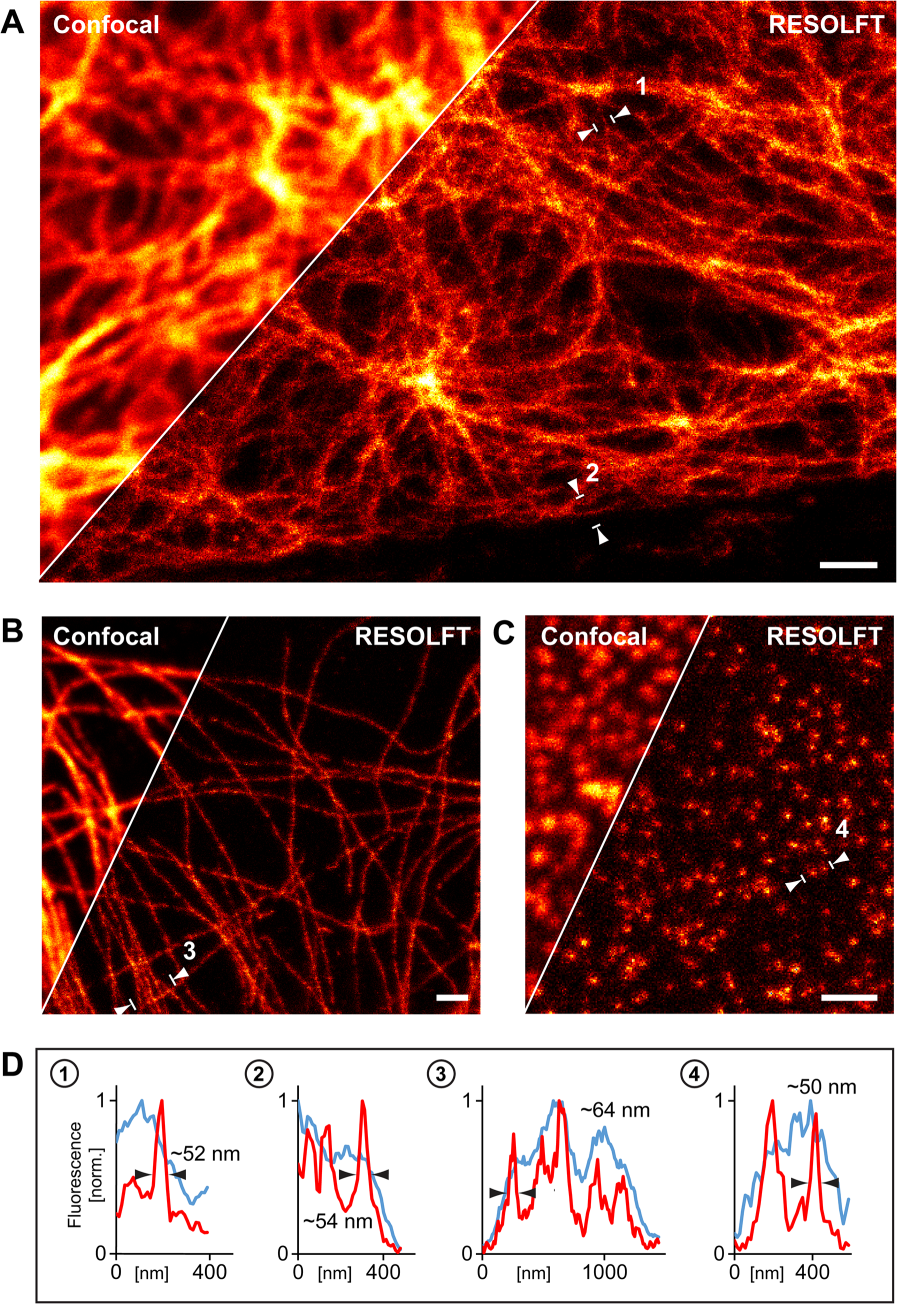
RESOLFT nanoscopy of methanol fixed cells. Comparison of RESOLFT super-resolution microscopy and the corresponding confocal microscopy images of CV-1 cells decorated with primary antibodies against vimentin (A), α-tubulin (B) and the nuclear pore complex protein Nup153 (C). (D) Line-profiles of the fluorescence intensities recorded between the arrowheads in (A-C), as indicated (confocal: light blue; RESOLFT: red). The line profiles in (1–3) are averaged across five adjacent line profiles that were perpendicular across the respective filament. The distance between two adjacent line profiles was the edge length of one pixel. Scale bars: 1 μm.

In order to image FLASR labelled fixed cells by RESOLFT super-resolution microscopy, we decorated methanol-fixed CV-1 cells with monoclonal antibodies against vimentin (Fig 2A), α-tubulin (Fig 2B) or the nuclear pore complex protein Nup153 (Fig 2C). After a washing step, the cells were incubated with purified FLASR (0.1 mg/ml) for 1 h. After further washing steps, the cells were mounted in an aqueous buffer (Tris-NaCl; pH 8.2). We chose this buffer as a mounting medium because in an aqueous buffered medium the switching properties of rsEGFP2 are preserved [10]. The pH of the buffer was set to 8.2 because the Z-domain has the highest affinity to IgGs at this value [20,21].

For imaging, we utilized a modified commercial point scanning RESOLFT setup. Overcoming the diffraction barrier in point scanning RESOLFT-type imaging requires the use of a specific light irradiation sequence at every point (for details see [9,10,22]). We found that the rsEGFP2 switching characteristics of the buffer embedded FLASR were comparable to those observed for rsEGFP2 expressed as a fusion protein in living cells. Hence we used the same imaging parameters as for live cell imaging. To record the FLASR labelled samples by RESOLFT microscopy, the rsEGFP2 proteins were first switched into the On-state by irradiation with light of 405 nm (2–5 μW, measured at the back focal plane of the objective) for 20–40 μs. Then, the proteins in the periphery of the focal spot were switched off with a doughnut shaped beam of 488 nm (17–20 μW) for 400–460 μs. Finally, the fluorescence was detected while exciting with a Gaussian shaped beam of 488 nm (3.4–8.2 μW) for 30–45 μs. This irradiation sequence was repeated at each scanning position. For the detailed imaging parameters, see S1 Table.

**Fig 3:**
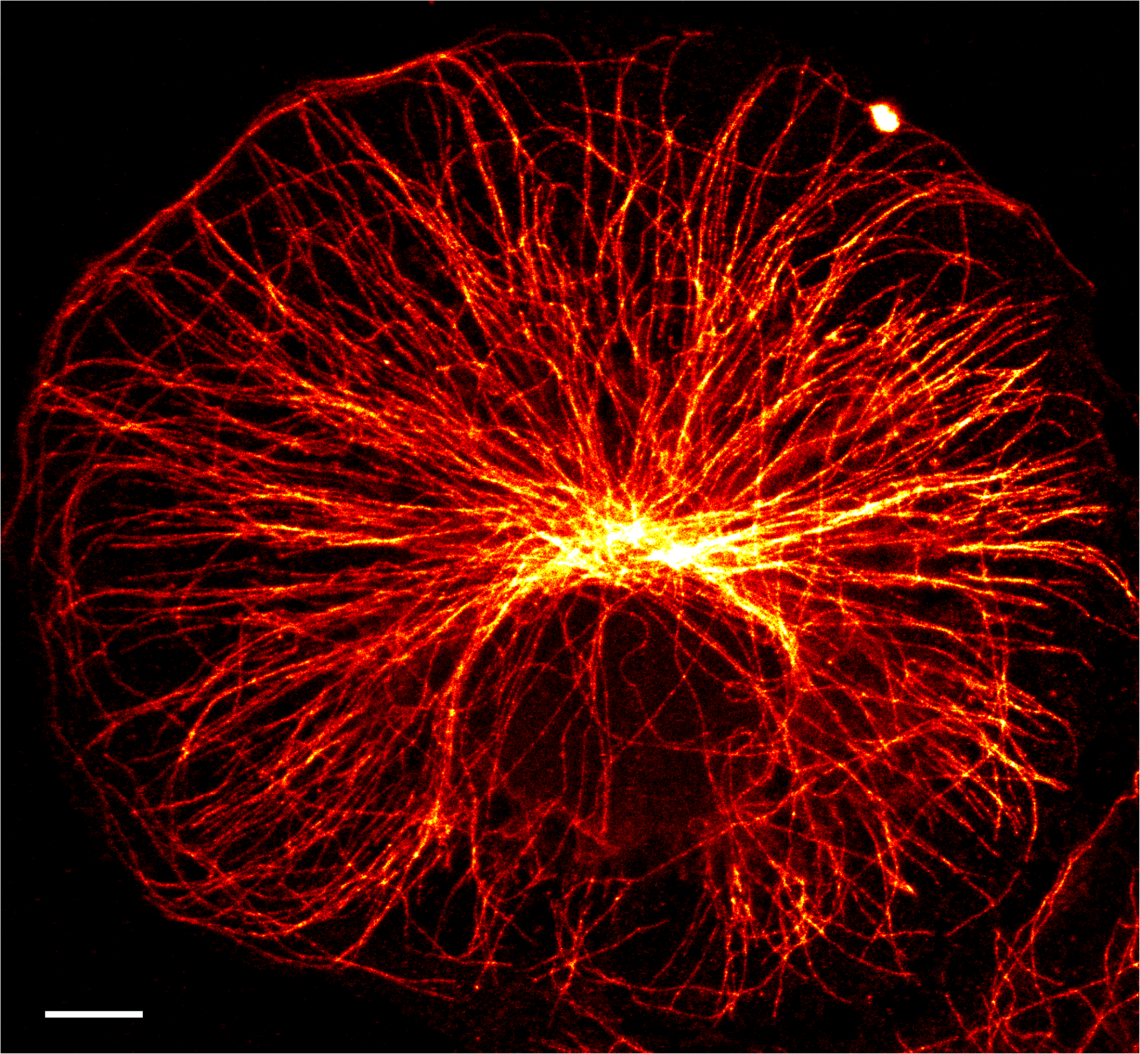
RESOLFT super-resolution image of an entire CV-1 cell. The cell was decorated with primary antibodies against α-tubulin and FLASR. Scale bar: 5 μm.

In all three samples, the RESOLFT approach revealed structural details that were indiscernible in the corresponding confocal images (Fig 2). In case of vimentin, averaging several adjacent line profiles across single thin vimentin filaments consistently revealed a full width at half maximum (FWHM) of < 55 nm. On microtubules, this value was on average slightly larger (< 65 nm), reflecting the fact that the diameter of single microtubules is larger than the diameter of thin vimentin filaments. Previous super-resolution studies using conventional immunofluorescence with organic dyes reported similar diameters of the antibody decorated filaments [23,24]. Importantly, adjacent structures, which are masked by the lower resolution in the diffraction-limited confocal images, are resolved in the corresponding RESOLFT images (Fig 2). Because of the lack of cellular motion, we were able to image an entire CV-1 cell in a field of view of 58 μm × 54 μm decorated with an antibody against α-tubulin in the RESOLFT mode (Fig 3).

Altogether, we conclude that FLASR-like probes are a suitable tool to label fixed cells for RESOLFT and other super-resolution techniques requiring (switchable) fluorescent proteins in their native state.

## Discussion

Previously, RESOLFT imaging was restricted to living cells expressing RSFP fusion proteins. With FLASR, RESOLFT can be extended to formaldehyde or methanol fixed samples. This opens up new applications for RESOLFT super-resolution microscopy, but also makes FLASR a tool for calibrating and testing the performance of a RESOLFT microscope without the inherent dynamics of living cells.

FLASR is a fusion protein consisting of the synthetic antibody-binding ZZ domain fused to two rsEGFP2 proteins. We successfully used FLASR to decorate chemically fixed cells labelled with various primary antibodies. The low FLASR dissociation rates (Table 1) from the Fc-region of the primary antibodies presumably account for the good staining efficiency and the high signal-to-noise ratio despite relatively low association rates. FLASR allows the labelling of chemically fixed cells while maintaining the fluorescent protein in an aqueous environment, thus leaving the switching characteristics of rsEGFP2 similar to the situation in a living cell.

**Fig 4:**
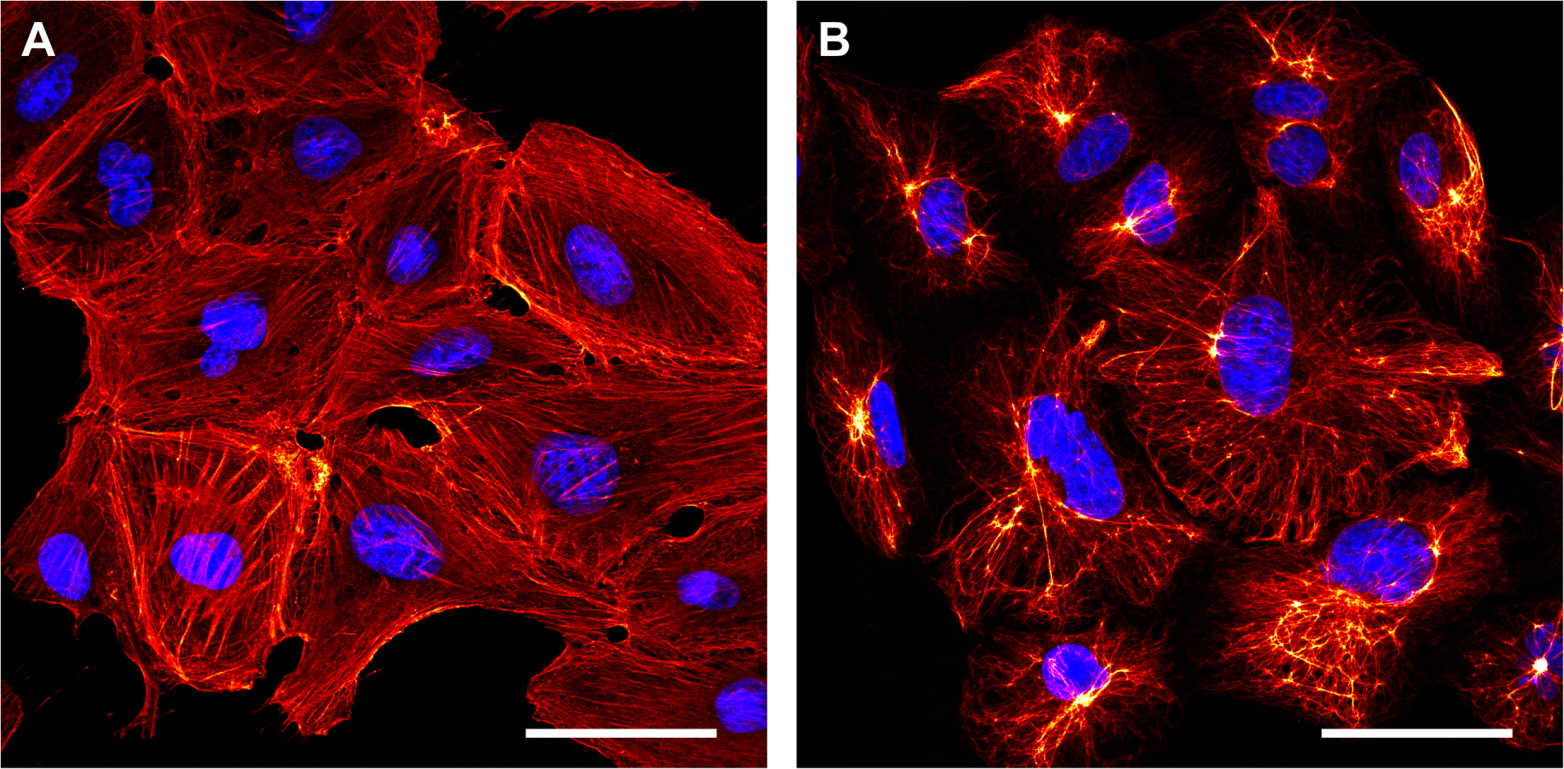
Immunolabelling with an M-rsEGFP2_tandem_ fusion protein. Maximum intensity projections of confocal microscopy z-stacks of methanol fixed CV-1 cells immunolabelled with antibodies against β-actin (A) and vimentin (B). The purified recombinant fusion protein M-rsEGFP2_tandem_ was used to decorate the primary antibodies (red). Nuclei were labelled with DAPI (blue). Scale bars: 50 μm.

Potentially, instead of the ZZ-domain, also other binding domains, including affibodies and other small binders, might be utilized for this approach. Recently, the immunoglobulin-binding protein M of *Mycoplasma genitalium* was shown to bind to IgGs of human and other mammalian species with high affinity [25]. Unlike the Z-domain, it interacts with the conserved areas of the variable region of the κ and λ light chains, but seems not to interfere with already established antibody-antigen interactions. To test if protein M can be used to generate a FLASR-like probe, we generated an M-rsEGFP2_tandem_ fusion protein and utilized it successfully for labelling, using the same protocol as with FLASR (Fig 4).

FLASR labelled cells are an excellent technical sample providing uniform labelling across many cells. The sample preparation is easy and quick, similar to standard immunostainings [26]. In FLASR labelled samples, imaging parameters and the photophysical properties of the RSFPs can be analyzed in depth. The obtained imaging parameters can immediately be transferred to live-cell RESOLFT imaging. This labelling strategy, which can be expanded to other (switchable) fluorescent proteins, may be useful also for other microscopy approaches that rely on the switching of fluorescent proteins in aqueous environments, including SOFI [27], PALM [28], and others.

## Material and Methods

### Plasmid constructs and cloning

#### FLASR

The tandem Z-domain was amplified by PCR from plasmid pEZZ18 (GE Healthcare, Little Chalfont, Buckinghamshire, England) (forward primer: GTACGGATCCCTTAAGGCGCAACACGATGAAGCCGTAG/ reverse primer: GTACGGTACCACCGGTGGCACCACTACCCGCGTCTACTTTCGGCGCCTGAG). The PCR product was cloned via BamHI and KpnI in the pQE50 (Qiagen, Hilden, Germany) expression vector resulting in pQE50-ZZ. The rsEGFP2 coding sequence was amplified by PCR from pQE31-rsEGFP2 [10] (forward primer: CTAGCTGCAGCCACTAGTGGTAGTGGTGCCATGGTGAGCAAGGGCGAG/ reverse primer: GATCAAGCTTttaGTGATGGTGATGGTGATGCTGCAGcttgtacagctcgtc catgc) adding a 6xHis-Tag to the C-terminus. The PCR product was cloned via PstI and HindIII in the pQE50-ZZ vector resulting in the plasmid pQE50-ZZ-rsEGFP2-His. In a next step rsEGFP2 was amplified (forward primer: GCTAACCGGTGTGAGCAAGGGCGAGGAG/ reverse primer: CGATACTAGTCTTGTACAGCTCGTCCATGCCG) and cloned via AgeI and SpeI in the plasmid pQE50-ZZ-rsEGFP2-His resulting in the plasmid pQE50-ZZ-rsEGFP2_tandem_-His.

#### Protein M

For expression of protein M, the amino acid sequence of the recombinant protein M TD [25] was reverse translated *in silico*, optimized for the *E*. *coli* codon usage and the cDNA was purchased from GenScript (GenScript, Piscataway, NJ, USA). The protein M cDNA was cloned via AflII and AgeI in the expression vector pQE50-ZZ-rsEGFP2_tandem_-His, replacing the ZZ-sequence resulting in the expression vector pQE50-M-rsEGFP2_tandem_-His.

### Protein expression and purification

For protein expression, the plasmids pQE50-ZZ-rsEGFP2_tandem_-His or pQE50-M-rsEGFP2_tandem_-His were transformed into the *E*. *coli* expression strain BL21-CP-RIL. The recombinant proteins were purified on Ni-NTA agarose columns (Quiagen, Hilden, Germany) according to the standard protocols. After elution, the purified fusion proteins were transferred into PBS buffer (137 mM NaCl, 2.7 mM KCl, 10 mM Na_2_HPO_4_, 1.8 mM KH_2_PO_4_, pH 7.4) using ultrafiltration columns (Vivaspin 20, 10.000 MWCO PES, Sartorius Stedim Biotech, Göttingen, Germany) and stored at -80 °C.

### Surface Plasmon Resonance

Binding experiments were performed on a Reichert SPR Biosensor SR7000DC (Reichert technologies, Buffalo, NY, USA). As a ligand, 6xHis-tagged FLASR was immobilized on a Ni2^++^ chelator sensorchip NiHC500m (XanTec bioanalytics, Düsseldorf, Germany) at 30 μl/min flow rate, to an average surface density of 4000 μRIU on the left channel (sample channel). The untreated right channel was used as reference channel. The following unconjugated polyclonal antibodies were used: AffiniPure Mouse Anti-Rat IgG, AffiniPure Rabbit Anti-Goat IgG, AffiniPure Sheep Anti-Mouse IgG, and AffiniPure Goat Anti-Rabbit IgG (all from Dianova, Hamburg, Germany). For each analyte, 10 concentrations ranging from 7.8 nm to 4 μM were injected. Twice as many buffer injections (buffer reference) were performed for each analyte concentration. All interaction experiments were performed at 20 °C, at a flow rate of 40 μl/min, in running buffer containing 50 mM HEPES pH 7.4, 150 mM NaCl and 50 μM EDTA. The obtained sensograms were double referenced (reference channel and buffer injections) and globally fitted using Scrubber 2.0 (BioLogic Software, Campbell, Australia).

### Immunofluorescence

For immunofluorescence, CV-1 cells were seeded on glass cover slips and fixed with cold methanol (-20 °C) for 5 minutes. The cover slips were washed briefly in PBS and then incubated in 10% bovine serum albumin (BSA) (10% w/v BSA in PBS 7.4) for 5 minutes. The cells were decorated with primary antibodies against α-tubulin (Sigma-Aldrich, St. Louis, USA), vimentin (Sigma-Aldrich), β-actin (Sigma-Aldrich), Nup153 (Abcam, Cambridge, UK) at RT in a wet-chamber. After one hour of incubation the cover slips were washed in Tris-NaCl buffer (100 mM Tris-HCl 150 mM NaCl, pH 8.2) for 5 minutes. The purified FLASR protein was used to detect the primary antibody in a concentration of 100 μg/mL in Tris-NaCl buffer. After 1 h the cover slips were washed in Tris-NaCl buffer, mounted in Tris-NaCl buffer on microscope slides and sealed with picodent twinsil silicone (picodent, Wipperfürth, Germany). For conventional (confocal) light microscopy samples were embedded in Mowiol containing the anti-bleaching reagent DAPCO [26] and DAPI for DNA staining (final concentration: 2.5 μg/ml; Sigma Aldrich).

### Microscopy

#### Conventional light microscopy

Confocal light microscopy was performed using a confocal microscope (Leica SP5, Leica Microsystems, Wetzlar, Germany) equipped with a 1.40 NA oil immersion objective (Leica HCX PL APO lambda blue 63x/1.40–0.60 Oil UV). The channels were discriminated using the sequential scanning mode (line by line). First, the DAPI signal was recorded using the 405 nm laser. Then the rsEGFP2 fluorescence was recorded by excitation with 488 nm. Except for contrast stretching and smoothing, no further image processing was applied.

#### RESOLFT nanoscopy

RESOLFT microscopy was performed using a modified “RESOLFT Quad P” microscope (Abberior Instruments, Göttingen, Germany) equipped with an Olympus UPlanSApo 100x/1.4 Oil objective lens. For RESOLFT imaging of FLASR samples, the following switching scheme was used: First, proteins were switched into the on-state with light of 405 nm. Second, rsEGFP2 proteins in the periphery of the focal spot were switched off using a 488 nm doughnut-shaped beam. Third, on-state fluorophores at the center of the spot were read out with a Gaussian shaped beam of 488 nm light. After on-switching and before fluorescence readout, a short illumination break of 5 μs was integrated into the switching scheme. The RESOLFT images were recorded with triple line averaging; hence the switching scheme was performed three times at each scanning position. The applied laser powers, switching times and scanning step sizes of all RESOLFT images are detailed in S1 Table. Corresponding confocal images were recorded by applying the same switching scheme as used for RESOLFT imaging without the illumination step for off-switching with the doughnut shaped beam.

## Supporting Information

S1 Fig Equilibrium Binding Isothermes for secondary antibodies binding to FLASR. FLASR was immobilized on a Ni^2+^-chelator sensor chip. Concentrations of 7.8 nM to 4.0 μM of the indicated IgG were used to monitor the relative response once equilibrium was reached at the end of the association phase.
(PDF)

S1 Table Imaging parameters used for RESOLFT microscopy. Light intensities were measured in the back focal plane of the objective.
(PDF)
