# Supplementary material for: RESOLFT Nanoscopy of Fixed Cells Using a Z-Domain Based Fusion Protein for Labelling
Source: PLoS One. 2015 Sep 16;10(9):e0136233. doi: 10.1371/journal.pone.0136233 (PMC4574475; doi:10.1371/journal.pone.0136233)
Supplement: S1 Fig — FLASR was immobilized on a Ni2+-chelator sensor chip. Concentrations of 7.8 nM to 4.0 μM of the indicated IgG were used to monitor the relative response once equilibrium was reached at the end of the association phase. (PDF) [file pone.0136233.s001.pdf]

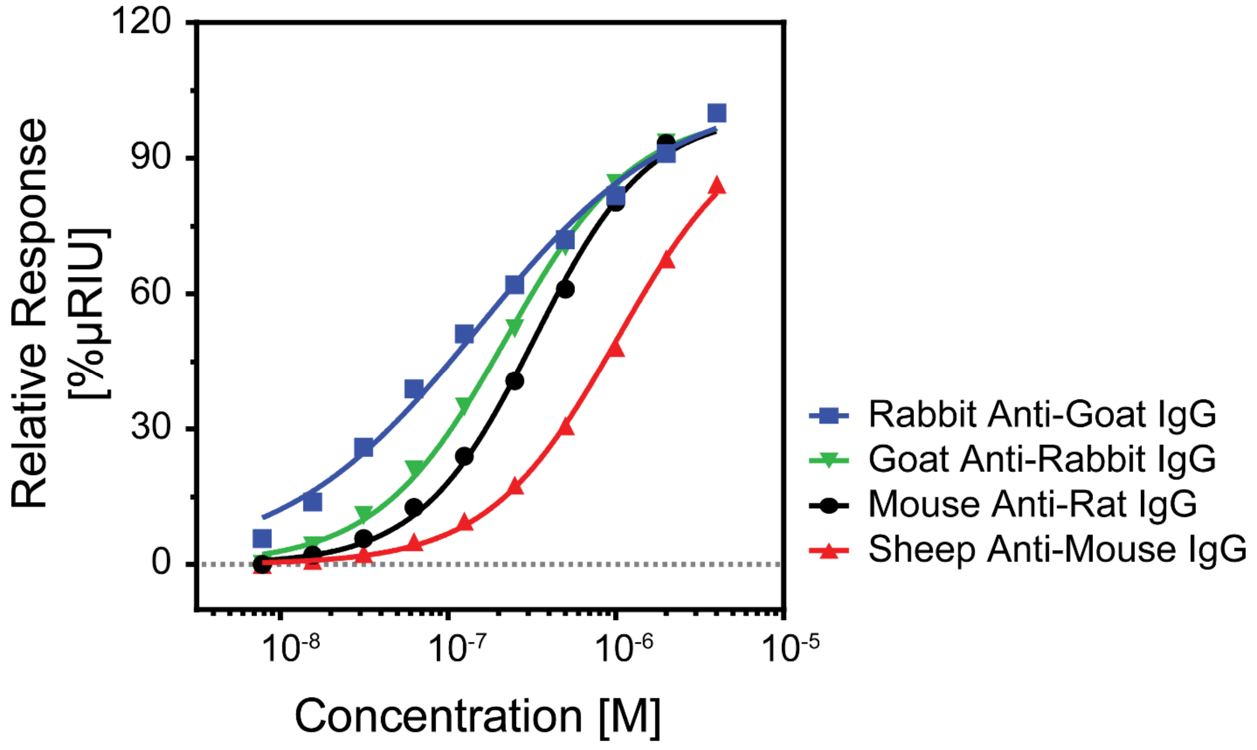

Suppl. Fig. 1. Equilibrium Binding Isothermes for secondary antibodies binding to FLASR. FLASR was immobilized on a Ni^{2+}-chelator sensor chip. Concentrations of 7.8 nM to 4.0 µM of the indicated IgG were used to monitor the relative response once equilibrium was reached at the end of the association phase.
