# Supplementary material for: RESOLFT Nanoscopy of Fixed Cells Using a Z-Domain Based Fusion Protein for Labelling
Source: PLoS One. 2015 Sep 16;10(9):e0136233. doi: 10.1371/journal.pone.0136233 (PMC4574475; doi:10.1371/journal.pone.0136233)
Supplement: S1 Table — Light intensities were measured in the back focal plane of the objective. (PDF) [file pone.0136233.s002.pdf]

Suppl. Table 1. **Imaging parameters used for RESOLFT microscopy.** Light intensities were measured in the back focal plane of the objective.

**RESOLFT image**	**405 nm on-switching**		**488 nm depletion beam**		**488 nm readout**		**scanning step size**
	power [µW]	*illumination time [µs]*	*power [µW]*	*illumination time [µs]*	*power [µW]*	*illumination time [µs]*	*[nm]*
Fig. 2a	2	20	17	430	8,2	30	20
Fig. 2b	2	20	17	430	7	40	25
Fig. 2c	5	20	17	460	3,4	40	20
Fig. 3	2	40	20	400	4,6	45	30
